# Environmental chemical exposures in the urine of dogs and people sharing the same households

**DOI:** 10.1017/cts.2020.548

**Published:** 2020-10-02

**Authors:** Kaitlyn Craun, Kristofer Ross Luethcke, Martin Shafer, Noel Stanton, Chen Zhang, James Schauer, Joshua Faulkes, Kaitlin E. Sundling, Daniel Kurtycz, Kristen Malecki, Lauren Trepanier

**Affiliations:** 1 Department of Medical Sciences, School of Veterinary Medicine, University of Wisconsin-Madison, Madison, WI, USA; 2 Wisconsin State Laboratory of Hygiene, University of Wisconsin-Madison, Madison, WI, USA; 3 Department of Pathology and Laboratory Medicine, School of Medicine and Public Health, University of Wisconsin-Madison, Madison, WI, USA; 4 Department of Population Health Sciences, School of Medicine and Public Health, University of Wisconsin-Madison, Madison, WI, USA

**Keywords:** Bladder cancer, chemical mutagens, sentinel, household exposure, one health

## Abstract

**Introduction:**

Urothelial carcinoma (UCC) develops in both humans and dogs and tracks to regions of high industrial activity. We hypothesize that dogs with UCC may act as sentinels for human urothelial carcinogen exposures. The aim of this pilot study was to determine whether healthy people and dogs in the same households share urinary exposures to potentially mutagenic chemical carcinogens.

**Methods:**

We measured urinary concentrations of acrolein (as its metabolite 3-HPMA), arsenic species, 4-aminobiphenyl, and 4-chlorophenol (a metabolite of the phenoxyherbicide 2,4-D) in healthy dogs and their owners. We assessed possible chemical sources through questionnaires and screened for urothelial DNA damage using the micronucleus assay.

**Results:**

Biomarkers of urinary exposure to acrolein, arsenic, and 4-chlorophenol were found in the urine of 42 pet dogs and 42 owners, with 4-aminobiphenyl detected sporadically. Creatinine-adjusted urinary chemical concentrations were significantly higher, by 2.8- to 6.2-fold, in dogs compared to humans. Correlations were found for 3-HPMA (*r* = 0.32, *P* = 0.04) and monomethylarsonic acid (*r* = 0.37, *P* = 0.02) between people and their dogs. Voided urothelial cell yields were inadequate to quantify DNA damage, and questionnaires did not reveal significant associations with urinary chemical concentrations.

**Conclusions:**

Healthy humans and pet dogs have shared urinary exposures to known mutagenic chemicals, with significantly higher levels in dogs. Higher urinary exposures to acrolein and arsenic in dogs correlate to higher exposures in their owners. Follow-up studies will assess the mutagenic potential of these levels *in vitro* and measure these biomarkers in owners of dogs with UCC.

## Introduction

Bladder cancer in humans is associated with environmental exposures [1,2]. Cigarette smoking is associated with about 50% of human bladder cancer cases [3], and occupational exposures from industrial processes or application of phenoxyherbicides and other pesticides account for an additional 2%–18% of cases [4–7]. At least one third of human bladder cancer risk may be due to chronic non-tobacco, non-occupational environmental exposures, but the role of specific toxic chemicals is not well understood.

Bladder cancer in humans is associated with regions of higher industrial activity, even among people in non-industrial occupations [5,8]. This may result from chronic low-grade exposures to known bladder carcinogens such as arsenic (found in contaminated ground water and air pollution), acrolein (found in air pollution and processed heat-treated foods), and 4-aminobiphenyl (found in cooking fumes and other sources of air pollution) [9,10]. However, estimating bladder cancer risk from specific environmental chemical exposures can be difficult because of the lengthy time for disease development. The time from environmental exposure to cancer development can be decades [5], and the median age of onset of urothelial carcinoma is 67 years [11].

Bladder cancer also develops in pet dogs, with an estimated incidence of more than 20,000 cases per year in the USA, and similar clinical and histopathologic features to human muscle-invasive urothelial cell carcinoma (UCC) [12,13]. The timeline for development of bladder cancer in dogs is compressed relative to humans, with a median age of onset of 8–11 years. Bladder cancer has not been associated with tobacco smoke exposure in dogs [14,15] and occupational exposures are unlikely. However, as in humans, UCC in dogs is associated with regions of higher industrial activity [8] and with household exposure to pesticides such as phenoxy herbicides [16].

Given similar disease manifestations and overlapping environmental risks, we hypothesize that UCC in humans and dogs is due to similar non-tobacco, non-occupational chemical exposures that lead to urothelial DNA damage. Our overall hypothesis is that dogs with UCC can act as sentinels for household urinary exposures to mutagenic bladder carcinogens in humans. Our specific hypothesis for this pilot study was that dogs and humans living in the same household would have evidence of shared urinary exposures to potential environmental bladder carcinogens, and that dogs would have higher concentrations due to their smaller body size and characteristic dog behaviors, such as grooming, rolling, sniffing, rooting, and digging in the soil.

The primary aim of this study was to compare urinary exposures to acrolein, inorganic arsenic, 4-chlorophenol (a soil metabolite of the phenoxyherbicide 2,4-D), and 4-aminobiphenyl in healthy dogs and their owners. Secondary aims were to assess associations with possible chemical sources through owner questionnaires and to evaluate accompanying urothelial DNA damage using the micronucleus (MN) assay in voided urinary cells.

## Materials and Methods

### Population Recruitment and Sample Collection

Dog owners were recruited by email from the population of faculty, students, and staff at the University of Wisconsin–Madison School of Veterinary Medicine. Recruitment, consent, and sampling procedures were approved by both the University of Wisconsin Institutional Review Board and the School of Veterinary Medicine Institutional Animal Care and Use Committee. Human subjects needed to be over 21 years of age and the primary caretaker of a dog sharing the same home for at least 1 year. Dogs could be of any age or breed, but without a cancer diagnosis and free of lower urinary tract signs within the past year.

Eligible dog owners were provided kits with standardized collection supplies and were instructed to collect 50 ml of morning urine from themselves and their pet dog on the same day. Only one person and one dog were sampled per household. Urine was allowed to settle in the refrigerator in a 250 ml conical polypropylene tube for 8 h. Five milliliter of urine sediment was then transferred from the bottom of the tube into ThinPrep^®^ vials (Hologic Inc., Marlborough, MA, USA) for cell fixation and subsequent MN assay. To accommodate 5 ml of urine volume, fixative volume was adjusted to 1 ml before inclusion in the kit. The voided urine and fixed urinary cells were hand delivered or shipped overnight on cold packs to the laboratory. Urine was frozen in multiple aliquots at −80°C within 24 h of collection and held at −80°C until immediately before biomarker analysis.

### Urinary Biomarker Assays

All chemical assays were performed on dedicated single aliquots of thawed, vortexed urine. Standards (and isotopically labeled internal standards, from CDN Isotopes, Quebec CA unless otherwise noted) were used to prepare 6–13 point calibration curves for all biomarkers, except arsenic species, in both human and canine urine.

#### Acrolein

Acrolein exposure was measured as its stable metabolite, 3-hydroxylpropyl mercapturic acid (3-HPMA), using an adaptation of a published LC-MS/MS method [17]. Calibration curves were prepared in 250 μl urine aliquots using authentic 3-HPMA, with d6-3-HPMA as an internal standard. Samples were assayed using a Sciex 4000 MS/MS interfaced with an Agilent 1260 HPLC; separation was achieved on a Phenomenex Luna 3 µm C8(2) 100 Å column with isocratic elution [0.5% acetic acid in water (90%) and acetonitrile (10%)]. Check standard recoveries ranged from 109% to 117%, and the LOQ (limit of quantitation) for 3-HPMA was 0.275 ng/ml (ppb).

#### Arsenic

Urinary arsenic was measured as total arsenic as well as major species: inorganic species As(III) and As(V), metabolites of inorganic arsenic [dimethylarsinic acid (DMA) and monomethylarsonic acid (MMA)], and non-toxic organic species (arsenocholine and arsenobetaine). Analysis was by coupled HPLC and magnetic-sector-inductively coupled plasma mass spectrometry (SF-ICP-MS). Standard stock solutions were prepared from arsenite [As(III)] and arsenate [As(V)] (from SPEX), and arsenocholine, arsenobetaine, DMA, and MMA (from WackoUSA, BOCsciences and SantaCruz Biotechnology). Urine samples (0.76 ml) were diluted with ammonium acetate buffer and measured directly; an internal standard (IS) of gallium in 0.5% ethanol/2% HNO_3_ was added in-line to the SF-ICP-MS. The analytical system consisted of a metal-free flow-path Shimazu HPLC interfaced to a magnetic sector ICPMS (Thermo Fisher Element XR), with a Hamilton PRP-X100 5 µm, 4.6 × 250 mm analytical column, and gradient elution with 100 mM ammonium carbonate/NH_4_OH, pH 9.4, from 0.62% to 95% over 18.5 min, at 27°C. The sum of the measured individual arsenic species accounted for, on average, 93% of the separately quantified total arsenic. Calibration check recoveries averaged 104% across all species, with LOQs of 0.02–0.09 ng/ml (ppb).

#### 4-chlorophenol

Exposure to the phenoxyherbicide 2,4-D was measured as its soil metabolite, 4-chlorophenol (4-CP) [18,19], which is mutagenic to mammalian cells *in vitro* [20]. Urine samples were assayed for 4-CP using a previously published LC-MS/MS method [21]. Urine samples (1.0 ml) were spiked with d4-4-CP as an internal standard and were acid hydrolyzed to release glucuronide conjugates. The LC-MS/MS system included a Sciex 4000 MS/MS interfaced with an Agilent 1260 HPLC. Separation in the LC was achieved on a Phenomenex Luna 3 µm C8(2) 100 Å column. Check standard recoveries ranged from 71% to 120%, with an LOQ of 0.50 ng/ml (ppb).

#### 4-Aminobiphenyl

4-Aminobiphenyl (4-ABP) was measured using an adaptation of a published method [22]. Urine (5 ml) was spiked with 4-ABP (Sigma Aldrich, St. Louis, MO, USA) to generate calibration curves; 4-ABP-d9 was used as an internal standard. Samples were acid hydrolyzed to release glucuronide conjugates and pH adjusted to 6.0. Sample cleanup was performed by solid phase extraction, using a PAHs (polycyclic aromatic hydrocarbon) MIPs SPE cartridge (SupelMIP SPE-PAHs 50 mg, 3 ml, SUPLCLE Corp., USA) conditioned with cyclohexane. 4-ABP was eluted with ethyl acetate, and the final extract was evaporated to dryness at 40°C and re-dissolved in methanol for LC-MS/MS analysis. Samples were analyzed on an Agilent 1260 HPLC coupled with a Sciex 5500 triple quad/Qtrap mass spectrometer; separation was achieved with a Waters Symmetry Shield RP18 (150 mm × 2.1 mm, 3.5 µm) column and gradient elution at 30°C. Check standard recoveries ranged from 80% to 120% with an LOQ of 0.005 ng/ml (ppb).

Urinary cotinine, a nicotine metabolite, was measured in all subjects to identify primary or second-hand smoke exposure [23]. Cotinine was measured from 10 μl of urine using a standard solid-phase competitive ELISA (Abnova Inc, Taiwan), with an LOQ of 5 ng/ml. All urinary biomarkers were normalized to urine creatinine (kinetic Jaffe method, QuantiChrom™Creatinine Assay Kit, Bioassay Systems, Hayward, CA, USA) to control for variable urine concentrations.

### Household Questionnaire

Owners also completed a self-administered questionnaire about everyday exposure to sources of known toxic chemicals, including household drive-by traffic, known manufacturing sites within a mile of the home, frequency of yard herbicide and pesticide use, drinking water sources, and tobacco usage at home (Supplementary file S1). Limited demographics (age by decade, race, and biologic sex) were obtained from the dog owner; age, breed, sex, neuter status, and body weight were collected for the dogs. Recent diet was queried for both species. Questionnaires and samples were encoded with a unique household study ID to protect owner confidentiality.

### Urothelial DNA Damage

Urine sediments preserved in ThinPrep^®^ vials were used for slide preparation using an automated processor (Hologic ThinPrep T-2000). Slides were Papanicolaou stained and screened using 100x and 400x magnification by a senior cytotechnologist (JF) to quantify urothelial and squamous cell DNA damage as micronuclei (MNi) per 2000 cells [24]; slides were reviewed by a cytopathologist (KS or DK) using standard morphologic criteria [25].

### Statistical Analyses

Urinary chemical concentrations, divided by urine creatinine, were compared between dogs and humans using unpaired, 2-tailed Mann–Whitney *U* tests. Chemical concentrations were correlated across human and pet dog pairs using Spearman’s rank correlation coefficients. Exploratory comparisons between urinary chemical concentrations in subjects with and without detectable MNi were made using Mann–Whitney *U* tests. Associations between self-reported household sources of chemical exposures and the highest versus lowest quartiles of urinary biomarker concentrations were examined using Fisher’s exact tests. *P* < 0.05 was considered significant. All analyses were performed using commercial statistical software (GraphPad Prism, GraphPad Software, San Diego, CA, USA). Multivariate analyses were not performed because of the small subgroup sizes.

## Results

### Population Demographics

Study kits were provided to 53 dog owners; urine samples and questionnaire data were returned from 42 households (80% response). Demographic data for the enrollees are shown in Table 1. Dog owners were primarily Caucasian women under the age of 40, and dogs were from a variety of breeds ranging in age from 1.5 to 16.5 years old and 4.0 to 53.4 kg body weight.

**Table 1.** Demographic data for 42 healthy pet dogs and their owners sharing the same household, which were surveyed for urinary exposures to environmental chemicals

Healthy pet dogs		Adult dog owners	
Median age (range)	5.0 years (1.5–16.5)	Age category (*n*, %)	22–30 years (19; 45%)
			31–40 years (11; 26%)
			41–50 years (5; 12%)
			51–60 years (6; 14%)
			61–70 years (1; 2%)
Sex (*n*, %)	Female (18; 43%)	Sex (*n*, %)	Female (33; 79%)
	Male (24; 57%)		Male (9, 21%)
Breed (*n*, %)	Mixed breed (23; 54%)	Race (*n*, %)	Caucasian (41; 98%)
	Labrador retriever (5; 12%)		Asian (1; 2%)
	Golden retriever (2; 5%)		
	Great Dane (2; 5%)		
	Viszla (2; 5%)		
	Indiv. purebreeds (8; 19%)		

### Urinary Biomarkers

The median urine volume submitted was 40 ml for dogs (range 5–175 ml) and 100 ml for human participants (range 35–250 ml). Urine creatinine was higher in dogs (median 204 mg/dl, range 35–263 mg/dl) compared to humans (median 90 mg/dl, range 15–176 mg/dl), as expected based on higher urine concentrating ability in dogs compared to humans [26,27].

**Fig. 1.**
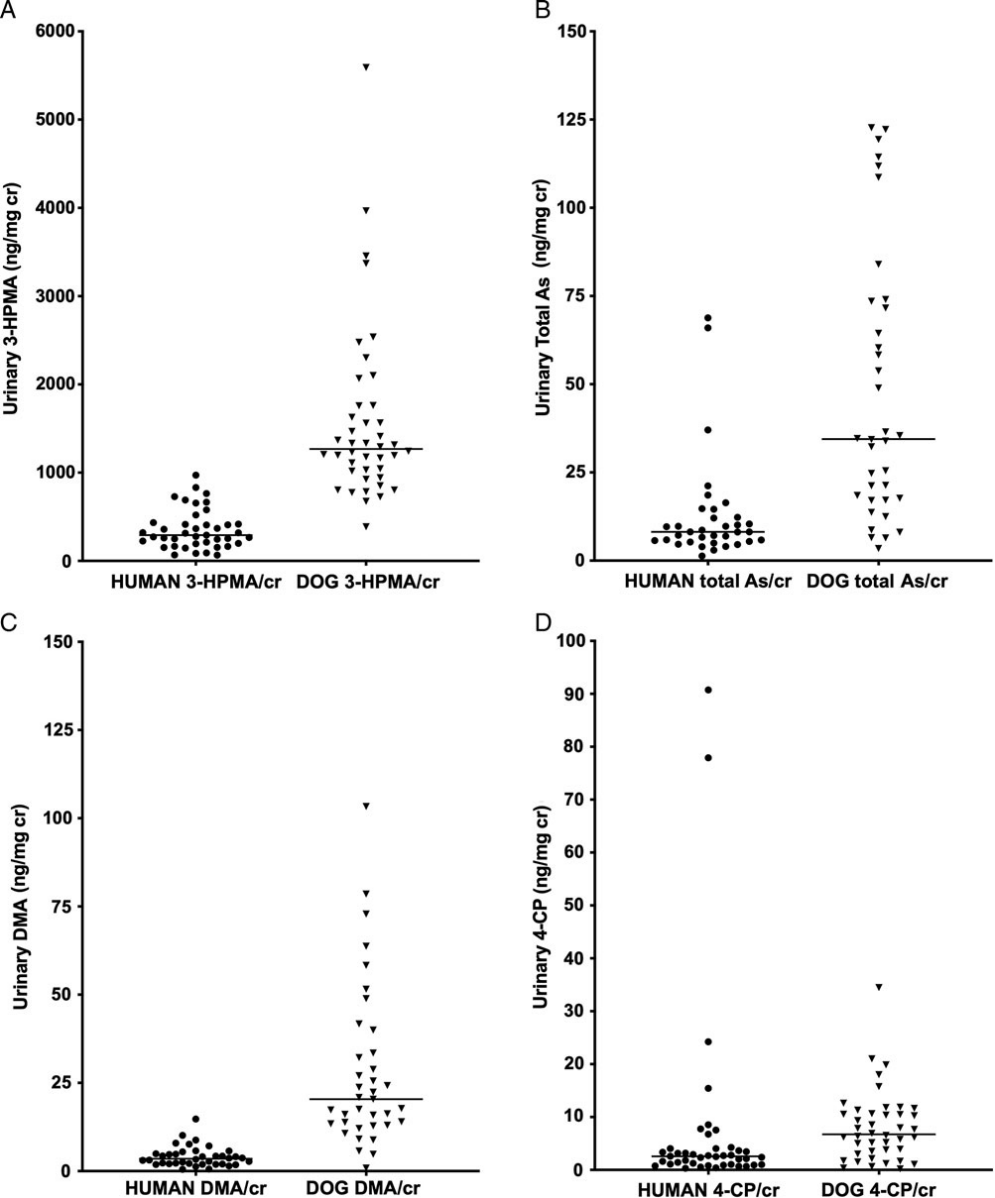
Urinary chemical concentrations in human subjects and their pet dogs sharing the same households. Shown are the acrolein metabolite 3-HPMA (panel A: *P* < 0.0001 between groups); total arsenic (panel B: *P* < 0.0001 between groups); the major inorganic arsenic metabolite dimethylarsinic acid (DMA; panel C: *P* < 0.0001 between groups), and 4-chlorophenol (4-CP), the soil metabolite of the phenoxyherbicide 2,4-D (panel D: *P* = 0.0004 between groups).

The acrolein metabolite 3-HPMA was quantifiable in all urine samples, with wide variability in concentrations among humans and dogs as adjusted for urine creatinine (Fig. 1A). Median 3-HPMA/cr concentrations were significantly higher in dogs (1269 ng/mg) compared to humans (285 ng/mg; *P* < 0.0001), and a median 4.9-fold higher between individual dogs and their owners (range from 0.6- to 24.8-fold).

Total urinary arsenic concentrations were significantly higher, by more than 4-fold, in dogs compared to humans (*P* < 0.0001; Fig. 1B**)**. The major urinary arsenic species in dogs and humans was dimethylarsinic acid (DMA), a metabolite that reflects exposure to inorganic arsenic [28]. Urinary DMA/cr concentrations were significantly higher in dogs (median 20.4 ng/mg) than in humans (median 3.5 ng/mg, *P* < 0.0001; (Fig. 1C), and a median of 6.2-fold higher between individual dogs and their owners (range from 0.7- to 29.2-fold). Concentrations of MMA were minor relative to DMA in both species (Supplementary file S2) but were significantly higher in humans (median 0.47 ng/mg) than in dogs (median 0.07 ng/mg; *P* < 0.0001).

Urinary levels of 4-CP/cr showed wide inter-individual variability within both species (Fig. 1D). Median concentrations were significantly higher in dogs (median 6.7 ng/mg) than humans (2.6 ng/mg; *P* < 0.0003). Observed 4-CP/cr concentrations were a median of 2.8-fold higher in individual dogs compared to their owners, but this ratio ranged widely, from 0.01- to 31.2-fold.

4-aminobiphenyl concentrations were below or near the LOQ in many canine and most human urine samples. The proportion of samples with detectable 4-aminobiphenyl, defined as ≥0.005 ng/ml when unadjusted for creatinine, was significantly higher in dogs (44.4%, range 0.001–0.011 ng/mg creatinine) than in humans (10.8%; range 0.002–0.026 ng/mg creatinine; *P* = 0.0016).

Cotinine was detected in only two human urine samples (1144 and 193 ng/mg cr); one of these had the highest measured urinary 4-aminobiphenyl concentration. Cotinine was detected in the urine of only one dog, belonging to the latter subject. This dog had the smallest body weight (4 kg) of the canine group.

### Correlations in Urinary Chemicals Between Humans and Dogs

**Fig. 2.**
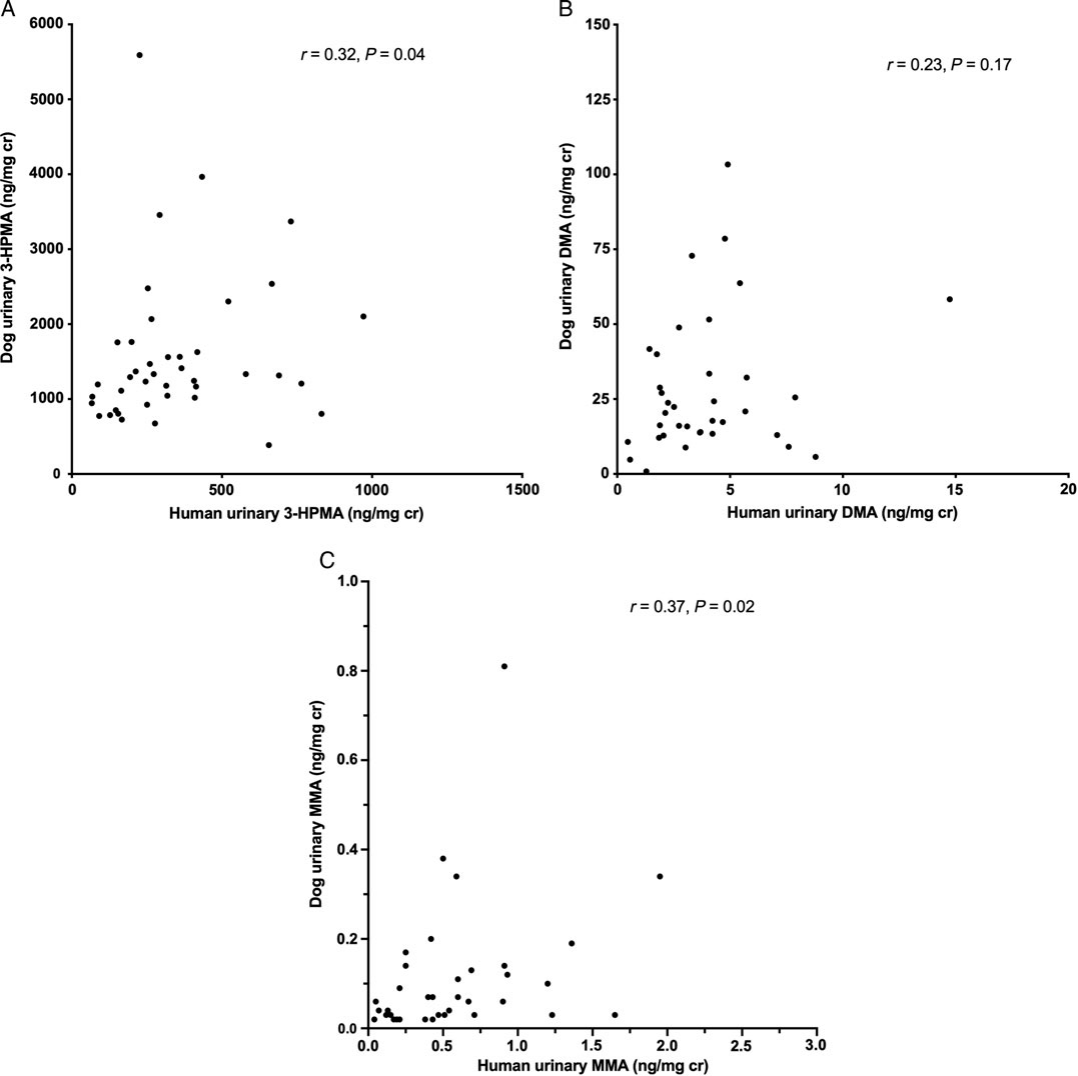
Correlations between urinary chemical concentrations in humans and pet dogs sharing the same households. Shown are the acrolein metabolite 3-HPMA (panel A: *r* = 0.32, *P* = 0.04); and the inorganic arsenic metabolites dimethylarsinic acid (DMA; panel B: *r* = 0.23, *P* = 0.17) and monomethylarsonic acid (MMA; panel C: *r* = 0.37, *P* = 0.02 for entire data set; one canine outlier (6.59 ng/mg) removed for graph resolution).

There was a significant positive correlation between urinary concentrations of 3-HPMA across paired human and dog samples from the same households (Spearman Rank *r* = 0.32, *P* = 0.04; Fig. 2). There was also a significant positive correlation between urinary MMA across dogs and their owners (*r* = 0.37, *P* = 0.02), but not for DMA (*r* = 0.23, *P* = 0.17) in this sample size. There was no correlation of 4-CP concentrations between dogs and humans (*P* = 0.79), even when two human outliers were excluded (*r* = 0.16, *P* = 0.32). None of the urinary chemical concentrations were correlated inversely with body weight across the dog population (*P* = 0.42–0.72).

### Household Exposures and Urothelial DNA Damage

Selected owner-reported household exposures are outlined in Table 2. Urinary concentrations of 3-HPMA/cr, DMA/cr, and 4-CP/cr in the top 50% for each species were compared to the bottom 50% for associations with owner-reported household exposures. High 3-HPMA/cr concentrations did not associate with household smoking in dogs or humans (*P* = 0.63, 0.99), but all dogs in households reporting “heavy” traffic past the house had urinary 3-HPMA/cr concentrations above 1000 ng/mg. High urinary 4-CP concentrations were not associated with household use of weed killer within the past year (*P* = 0.99, 0.99) or reported proximity to a golf course (*P* = 0.52, 0.51); however, we did not have household addresses to confirm this latter finding. Similarly, urinary DMA/cr was not associated with a treated lumber deck on the property (*P* = 0.47, 0.47).

**Table 2.** Selected environmental questionnaire data reported from 42 households that were screened for urinary chemical exposures in pet dogs and their owners

Exposure	Questionnaire response (*n*; %)
Traffic by home	Minimal (7; 17%)
	Moderate (30; 71%)
	Heavy (5; 12%)
Golf course within 1 mile	No (25; 60%)
	Yes (14: 33%)
	No response (3; 7%)
Insecticide use within past year	No (22; 52%)
	Yes (20; 48%)
Herbicide use within past year	No (17; 40%)
	Yes (25: 60%)
Drinking water	Municipal or bottled (34; 81%)
	Filtered well water (5; 12%)
	Unfiltered well water (3; 7%)
Treated lumber deck	No (19; 45%)
	Yes (13; 31%)
	Don’t know (10; 24%)
Tobacco smoking on property	No (29, 69%)
	Yes (13; 31%)

Urinary cell yields were generally poor from urine collected at home without centrifugation, with a median of only 100 urothelial cells per slide; only two samples from each species met ideal adequacy criteria for MN assays, containing at least 2000 urothelial cells per slide [24]. MNi were not detected in voided urothelial cells from any canine or human urine sediments. Some slides contained predominantly squamous cells, which contained MNi-like objects in 5 of 42 human samples. There was no discernable association between positive MNi-like objects in squamous cells and urinary chemical concentrations.

## Discussion

Biomarkers of urinary exposure to the mutagenic chemicals acrolein, inorganic arsenic, 4-chlorophenol, and, to a lesser extent, 4-aminobiphenyl were found in the urine of healthy pet dogs and their owners, and urinary exposures were significantly higher in dogs. This may be due to canine behaviors such as sniffing, chewing, rolling, grooming, and grass eating, as well as spending more daytime hours in the home than working owners. Furthermore, urinary concentrations of the major metabolites of acrolein and arsenic were correlated between dogs and humans in the same homes, suggesting shared household exposures.

We measured urinary acrolein exposures in our study because acrolein initiates bladder cancer in rodents, transforms human urothelial cells *in vitro*, and leads to DNA adducts that are over-represented in human urothelial carcinomas compared to normal bladder tissue [29–31]. Exposure to acrolein was measured as its stable metabolite 3-HPMA [32]. Levels detected in humans in our study (median 280 ng/ml, unadjusted for creatinine) were comparable to those previously reported in non-smokers (219–420 ng/ml) [17,33,34]. Concentrations of 3-HPMA were more than 4-fold higher in dogs, which could be due to smaller body size, higher physical exposures, or species differences in acrolein conjugation to glutathione, either enzymatically or non-enzymatically, to generate 3-HPMA [35].

Potential household sources of acrolein, other than tobacco smoke, include inhalation of indoor emissions from frying foods in oils, and outdoor air pollution from automobile exhaust, wood fires, or industrial emissions [35]. Oral exposure through shared foods is a possible source [36], although most households reported that their dogs consumed primary commercial dog food without sharing “human food” with their owners.

In addition to total arsenic, we measured major inorganic and organic arsenic species, with a focus on inorganic arsenic, which is carcinogenic, and its methylated metabolites DMA and MMA, which are classified as possibly carcinogenic [28]. DMA was the most prevalent urinary arsenic species in both humans and dogs, with more than 6-fold higher concentrations in dogs compared to humans. Canine hepatocytes are more efficient than human hepatocytes at converting inorganic arsenic to DMA [37], which could have had contributed to this difference. However, total arsenic concentrations were still 4-fold higher in dogs, indicating higher urinary arsenic exposures in dogs independent of metabolic interconversions. Conversely, urinary MMA concentrations, although a relatively minor component, were significantly higher in humans compared to dogs, which is also consistent with *in vitro* data showing more efficient MMA generation in human versus canine hepatocytes [37]. Interestingly, urinary MMA, but not DMA, positively correlated between dogs and their owners. Both metabolites are generated by the same enzyme, arsenic methyltransferase, in humans and dogs [37], and it is possible that we were underpowered to detect a correlation for DMA.

The phenoxyherbicide 2,4-D has been associated epidemiologically with bladder cancer in both humans and dogs [7,16]. However, 2,4-D itself is not a demonstrated mutagen [38]. Therefore, we measured a major soil metabolite, 4-chlorophenol, which is mutagenic to human cells *in vitro* [20]. Median concentrations of 4-CP found in human urine in our study (2.5 ng/ml unadjusted) were comparable to average concentrations previously reported in healthy human populations (1.1–1.5 ng/ml) [39–41]. Concentrations of 4-CP were significantly higher in dogs in our study than in humans, although there was a wide range of urinary exposures and no correlation between dogs and their owners. We observed two human urine samples with outlier increased 4-CP concentrations; both reported use of pesticides at home but did specify the types. Besides microbial degradation of 2,4-D in soil, other sources of 4-CP include surface and ground waters, chlorination byproducts in drinking water, and industrial effluents from paper mills [42].

As for 4-ABP, we found low to undetectable concentrations in most canine and human urine samples, with the highest concentration observed in a cotinine-positive smoker. This is consistent with previous studies of urinary 4-ABP in smokers versus non-smokers [22,43,44]. The higher proportion of dogs versus humans with detectable 4-ABP could be attributable to smaller body size, although we did not detect a correlation between body weight and 4-ABP concentrations in dogs. Potential non-tobacco, non-occupational sources of 4-ABP exposure include cooking oil emissions [45], food dyes [46], and even some hair dyes [47] (the latter being an unlikely route of exposure for dogs).

We normalized all urinary analytes to urine creatinine to control for individual and species differences in urine concentration. Median urine creatinine was higher in dogs, which is consistent with the dog’s higher urine concentrating ability compared to humans [26,27]. This normalization should not have affected our conclusions because correction for creatinine should lead to lower concentrations in dogs relative to humans, not the relatively higher concentrations that we observed. However, urine creatinine in people can vary by sex, age, and ethnicity [48], and while this should have been a minor variable in our population of mostly Caucasian women, urine specific gravity should be incorporated as an additional normalization factor in future studies.

Our questionnaire was not able to elucidate potential chemical sources in subjects with the highest quartiles of urinary chemical concentrations. We observed that some respondents were unsure about what specific chemicals were used on their lawn, how their drinking water was treated or filtered, and whether there were specific industries within a mile of their home. Our findings were also constrained by a relatively homogenous human population from a limited number of zip codes. Subsequent studies should recruit nationally and beyond a population of academic employees, objectively map household addresses to nearby chemical industries, and incorporate direct environmental sampling of the household.

The highest detected urinary concentrations of 3-HPMA (12,300 ng/ml in dogs and 984 ng/ml in humans, both uncorrected for creatinine) exceeded acrolein concentrations shown to be mutagenic to human urothelial cells *in vitro* (2.5 µM, equivalent to 550 ng/ml 3-HPMA) [30]. In addition, the highest detected DMA concentration in dogs (298.6 ng/ml uncorrected) also exceeded mutagenic concentrations demonstrated in human urothelial cells (1.25 µM, equivalent to 173 ng/ml DMA). However, we had inadequate urothelial cell yields to detect *in vivo* DNA damage in our study. We chose the MN assay because it has been shown to detect urothelial DNA damage in previous studies of smokers, industrial workers, and those exposed to arsenic in drinking water [24,49,50], and 50 ml of urine should have been sufficient to detect MN in our predominantly female population [51]. Despite this, we had poor urothelial cellularity in most samples, likely because we asked owners to pipette urine at home to obtain fresh fixed urine sediments. In addition, the MN assay requires cell turnover to manifest DNA damage as cytologically visible DNA fragments [24], and cell turnover is relatively slow in urothelial cells [52]. Methods that detect real-time DNA damage independent of cell division, such as the γ-H2AX assay that measures phosphorylated histones [53], along with an alternative method of urine sediment collection, are planned for follow-up studies.

The presence of large numbers of squamous cells in some of the samples was a potential confounding factor. Squamous cells may arise in the bladder trigone, but they may also be contaminants from the external urethra, in both males and females, or the vulva in females. Thus, the significance of MNi within squamous cells is unclear.

The strengths of this study include the use of paired human and canine samples from the same households and the targeted examination of structurally dissimilar mutagenic compounds associated with bladder cancer in both species. Limitations include a small sample size and heterogeneity among human and canine participants. However, despite these variables, we were still able to demonstrate correlations between pet dogs and their human owners for urinary exposures to acrolein and arsenic. We also had poor urothelial cell yield for DNA damage assessment and inexact wording in some parts of our questionnaire. These limitations will be addressed in our upcoming study of dogs with UCC and their owners.

Overall, we found that urinary exposures to acrolein, inorganic arsenic, and 4-chlorophenol, a mutagenic metabolite of the herbicide 2,4-D, were common in dogs and their owners sharing the same household. Concentrations of these chemicals were significantly higher in pet dogs, which could be due to increased physical exposure as well as spending more daytime hours in the home than their working owners. Urinary exposures to both acrolein and arsenic metabolites were correlated between dogs and their owners, suggesting shared household exposures, and reached concentrations predicted to be mutagenic in some individuals. Ongoing work will test the hypothesis that the observed levels of urinary chemical exposures directly lead to early urothelial DNA damage *in vitro* and will assess these urinary biomarkers in a case-control study of dogs with urothelial cell carcinoma along with their owners.

## Supplementary material

For supplementary material accompanying this paper visit https://doi.org/10.1017/cts.2020.548. click here to view supplementary material
