# Supplementary material for: Environmental chemical exposures in the urine of dogs and people sharing the same households
Source: J Clin Transl Sci. 2020 Oct 2;5(1):e54. doi: 10.1017/cts.2020.548 (PMC8057441; doi:10.1017/cts.2020.548)
Supplement: Supplementary file 1 [file S2059866120005488sup.zip › S2059866120005488sup001.docx]

**Supplemental file S2**: Arsenic subspecies found in the urine of 42 pet dogs and 42 dog owners. Data are reported as medians with observed ranges, and are shown as measured, uncorrected for urine creatinine. Levels below the limit of detection were encoded as one unit below the limit of detection.

| **Arsenic species** | **Dog urine** | **Human urine** |
| --- | --- | --- |
| Dimethylarsinic acid  (DMA; ng/mL) | 29.35  (0.06^1^-298.6) | 2.56  (0.56-24.25) |
| Monomethylarsonic acid (MMA; ng/mL) | 0.06^1^  (0.06-14.9) | 0.35  (0.06^1^-2.07) |
| Arsenic (III)  (ng/mL) | 0.01^1^  (0.01-0.25) | 0.01^1^  (0.01-6.13) |
| Arsenic (V)  (ng/mL) | 0.43  (0.02^1^-11.9) | 0.12  (0.02^1^-1.11) |
| Arsenocholine  (ng/mL) | 0.08^1^  (0.08-6.65) | 0.08^1^  (0.08-0.23) |
| Arsenobetaine  (ng/mL) | 0.08^1^  (0.08-260.32) | 0.48  (0.08^1^-84.02) |
| Total arsenic  (ng/mL) | 50.43  (2.16-369.51) | 5.13  (0.86-88.93) |

^1^Below the limit of detection
