# Supplementary material for: Environmental chemical exposures in the urine of dogs and people sharing the same households
Source: J Clin Transl Sci. 2020 Oct 2;5(1):e54. doi: 10.1017/cts.2020.548 (PMC8057441; doi:10.1017/cts.2020.548)
Supplement: Supplementary file 1 [file S2059866120005488sup.zip › S2059866120005488sup002.docx]

## **Household Environment Questionnaire**

The University of Wisconsin-Madison, School of Veterinary Medicine is conducting a study of environmental chemical exposures in pet dogs and their owners.

Along with obtaining a urine sample from you and your dog, we are asking you to complete this questionnaire about you and your household. All information collected will be kept confidential and will not be linked to you or your family in any publications.

PLEASE DO NOT PUT YOUR NAME ON THE SURVEY! This is to help to protect your privacy.

#### General Information about you

- Age in years ____22-30 ____31-40 ____41-50 ____ 51-60 ____61-70 ____71+
- Biologic sex _____ Male ____ Female
- Race

____ Caucasian (white)

____ Black or African American

____ Asian

____ Native American

____ Other

- Occupation(s) over the past year _________________________________________
- Have you ever worked in an industrial or factory environment? ______Yes _____No

If yes, please explain ___________________________________

- Which of the following foods have you eaten in the 24 hours prior to taking your urine sample – (check all that apply):

____ Shrimp or crab

____ Sushi with seaweed wrap

____ Tuna, salmon, sardines, or swordfish

____ Rice

____ Cereal

____ Roasted coffee

____ Beer or wine

____ Food fried in oil (potato chips, french fries, meat, tofu...)

____ Broccoli, cauliflower, brussels sprouts, or kale

____ None of the above

#### General Information about your dog

- Age of your dog _______________ (the one providing urine for this study)
- Sex of this dog _________
- Breed of this dog ____________
- Approximate weight in pounds ______________
- How many years have you owned this dog? ___________
- Have you and this dog lived in your present home for at least one year? ___________
- My dog’s diet is predominantly (more than 75% of intake):

_____ Dry kibble

_____ Canned dog food

_____ Raw food

_____ Home cooked diet

_____ “People” food

_____ Other ___________________________

- My dog’s typical brand of dog food is: ____________________________________

____________________________________________________________________

- Has this dog been treated with chemotherapy drugs for any type of cancer within the past 6 months (excluding prednisone)? ______________________________________________________

1. **Information about your household**

- Zip code of your home _____________
- Approximate year your house was built (if known) _____________________
- Do you live on a farm” Yes No Other _______________________
- How would you characterize truck and automobile traffic past your house and yard? (check one)

Minimal (dead end or country road)

Moderate (suburban neighborhoods and local roads)

_______ Heavy (main thoroughfare or highway)

- Are you aware of any of the following located within a mile of your home?

Chemical plant: _____Yes _____No _____Don't know

Municipal dump: _____Yes _____No _____Don't know

Landfill: _____Yes _____No _____Don't know

Coal plant: _____Yes _____ No _____Don't know

Incinerator: _____Yes _____No _____Don't know

Farm: _____Yes _____No _____Don't know

Golf course _____Yes _____No _____Don't know

- In the past year, which of the following products have been used in the house or on your property? (check all that apply)

Insecticides (ant, wasp, termites), applied by homeowner

______ Times per year

______ Brand, if known

Commercial pest control treatments (Insecticides for ant, wasps, termites)

______ Times per year

______ Brand, if known

_______Weed killer, applied by homeowner

______ Times per year

______ Brand, if known

Commercial weed killer treatments

______ Times per year

______ Brand, if known

- What type of drinking water do YOU drink in your home?

Municipal water, no additional treatment

Municipal water with additional filtration (Britta, Culligan etc; does not include a water softener)

Well water, untreated

Well water, treated

______Filtration ______Distillation ________Disinfection _______Do not know

Bottled drinking water

______ Other __________________

- What type of drinking water does YOUR DOG drink in your home?

Municipal water, no additional treatment

Municipal water with additional filtration (Britta, Culligan etc; does not include a water softener)

Well water, untreated

Well water, treated

______Filtration ______Distillation ________Disinfection _______Do not know

Bottled drinking water

______ Other __________________

- Does your home have a porch or deck built from treated lumber?

Yes No Do not know

- Are you a tobacco smoker? Current Former (weeks since last cigarette: ______) Never Comments____________________________
- Do any members of your household or visitors smoke cigarettes, pipes or cigars in your home?

Yes No

If yes, approximate number of cigarettes/cigars/pipes per week ____________

- Do any members of your household or visitors smoke cigarettes, pipes or cigars on your property but outside the house?

Yes No

If yes, approximate number of cigarettes/cigars/pipes per week ____________
